# Influence of Stainless Needle Electrodes and Silver Disk Electrodes over the Interhemispheric Cerebral Coherence Value in Vigil Dogs

**DOI:** 10.3390/s18113990

**Published:** 2018-11-16

**Authors:** Mihai Musteata, Denis Gabriel Borcea, Raluca Ștefănescu, Gheorghe Solcan, Radu Lăcătuș

**Affiliations:** 1 Neurology Clinical Unit, Clinics Department, Faculty of Veterinary Medicine, University of Agricultural Sciences and Veterinary Medicine Ion Ionescu de la Brad Iasi, 700489 Iași, Romania; raluca.stef@yahoo.ro; 2 Internal Medicine Unit, Clinics Department, Faculty of Veterinary Medicine, University of Agricultural Sciences and Veterinary Medicine Ion Ionescu de la Brad Iasi, 700489 Iași, Romania; borceadega@yahoo.com; 3 Faculty of Veterinary Medicine, University of Agricultural Sciences and Veterinary Medicine Cluj-Napoca, 400372 Cluj-Napoca, Romania

**Keywords:** electroencephalography, electrodes, dog

## Abstract

Electroencephalography (EEG) is an objective diagnostic tool in the evaluation of cerebral functionality, both in human and veterinary medicine. For EEG acquisition, different types of electrodes are used, as long as they have no impact on the recorded background activity. However, to date, the influence of electrode type on quantitative EEG and cerebral coherence has not been investigated. Twenty EEG traces (ten with needle electrodes and ten with disk electrodes) were recorded from ten mesocephalic vigil dogs in a monopolar montage. Values for interhemispheric coherence for each frequency band were compared between stainless needle and silver disk electrodes traces. Our results show that the values of interhemispheric coherence in vigil dogs are depending of the type of electrodes used in EEG recordings. In the frontal (FP) channel, for delta and theta frequency bands, the registered coherence is significantly higher when stainless needle electrodes are used. Our results might have important consequences in the field of canine neurology and applied neuroscience, as the frontal channel analysis is preferred in aging and behavior studies.

## 1. Introduction

Electroencephalography (EEG) is an essential diagnostic tool in the evaluation of cerebral disorders [1,2,3,4]. It is widely used in establishing a diagnosis of epilepsy [5,6,7], but also in assessing the efficacy of anticonvulsivants or other drugs. In a clinical environment, EEG traces are analyzed visually [8]. In addition, quantitative EEG (qEEG) may offer an in-depth look at different frequency bands, making it possible to further appreciate brain coherence (the normalized linear correlation which exists between two pairs of EEG channels and informs the frequency components common to them). From a mathematical point of view, coherence is calculated with the following formula:

$$Coh(f)=\frac{{\left|\sum_{i=1}^{N}F_{1}\left(f\right)\cdot F_{2}^{*}\left(f\right)\right|}^{2}}{\sum_{i=1}^{N}{\left|F_{1}\left(f\right)\right|}^{2}\cdot \sum_{i=1}^{N}{\left|F_{2}\left(f\right)\right|}^{2}}$$

where *Coh*(*f*) is the coherence function, *f* is frequency, *N* is the number of EEG realizations involved in averaging, *F*_1_(*f*) and *F*_2_(*f*) are Fourier transforms of EEG signals in two different channels, and * denotes a complex conjugation.

In humans, intra- and interhemispheric coherence is extensively studied in normal and pathological situations [9,10,11,12,13,14,15]. In veterinary medicine, due to the lack of an optimal protocol for EEG recordings [7], there are only few reports about cerebral coherence in healthy dogs or individuals with behavior problems, qEEG being mainly used in conditioning studies [14,16,17].

To record EEG traces, different types of electrodes are used [18,19,20]. Their usage is directly related to the type of investigation [21]. In human medicine, disk or cup electrodes are classically used, and those with a central hole allow periodic refilling with electrode conductive gel [22]. Needles are inserted just beneath the skin, parallel to the surface of the scalp and should be secured with an adhesive paste. Usually, they are not recommended for vigil patients or for prolonged recording, but have the advantage of being easy to place and may be used for short-term EEG in some comatose patients [22]. In veterinary medicine, the most used electrodes are the subdermal needle and disk electrodes. Subdermal needle electrodes have the advantages that they are easy to place, are minimally invasive, and are more suitable for short-term EEG (less than 30–40 min). Their disadvantages are related to a higher occurrence of muscle artifact registration in comparison with the disk electrodes, as well as the risk of their displacement from the area of interest, especially in non-sedated patients. Disk electrodes can be made from tin, pure silver, pure silver with a gold coating, silver-silver chloride, or gold. They are not invasive and are attached to the skin with a paste that has good electrical conductivity [23]. Disk electrodes allow a prolonged EEG recording (even in vigil dogs), but the time of acquisition is directly influenced by the properties of the conductive paste. In sedated dogs, the EEG traces are characterized by a significant reduction in artifacts, but the differences among the electrodes are not considered clinically relevant [23]. However, in this study, the authors focused on the impacts of electrodes over the visual background activity and the presence of artifacts; however, no qEEG assessment was performed. The disk electrodes are able to acquire the biopotentials from a wider cortical area compared to the needle electrodes. When the brainstem auditory evoked potentials were assessed using disk electrodes, Musteata (2013) observed that the brainstem latencies were shorter in comparison with those obtained when needle electrodes were used [24]. Because disk electrodes record electrical impulses of a higher number of neurons than the needle electrodes, it is possible to have a different distribution of waves in the different frequency bands and different values for cerebral coherence. To our knowledge, there is no previous study in which the value of dog cerebral coherences are evaluated in relation to the type of electrode used.

The goal of this study is to analyze the interhemispheric cerebral coherence values in vigil dogs when needle and disk electrodes are used.

## 2. Materials and Methods

The study was conducted at the Neurology Department of the Veterinary Teaching Hospital (VTH) of Faculty of Veterinary Medicine of Iasi, Romania. Ethical approval for the study was obtained from the Ethics Committee of the Faculty of Veterinary Medicine, University of Agricultural Sciences and Veterinary Medicine “Ion Ionescu de la Brad” of Iași (No. 424/05.05.2018).

### 2.1. Animals

The study was performed on ten mesocephalic dogs (6 females and 4 males) referred to VTH Iași. For all patients, a general physical examination, followed by a neurological one, was performed. All dogs were free of any intracranial pathology (based on the absence of any cognitive impairment, deficits in cranial nerves and no changes in behavior). The dogs were aged between six months and four years.

### 2.2. EEG System and Electrodes

Electroencephalographic examinations were performed with an 8-channel digital system, “Neuron-spectrum-1” (Neuron-Spectrum.net, Neurosoft Ltd., Ivanovo, Russia) and the registered traces were analyzed using the corresponding software.

For EEG trace acquisition, two types of electrodes (disk and needle) were used: Disk electrodes (pure silver) with a 10-mm diameter and 1.5-mm touch proof connector (NE134A, Nihon Kohden, Tokyo, Japan), and 0.22 × 22.5 mm and 1.5 mm proof connector subdermal electrodes (stainless steel) (NE-224S EEG, Nihon Kohden, Tokyo, Japan).

The parameters used for each electroencephalographic recording were: sensitivity, 10 μV; time constant, 0.3 s; filter pass-down of 35 Hz; filter pass-up 0.5 Hz; and electrode impedance <10 Ω, notch filter active.

### 2.3. Experiment Protocol

Before EEG acquisition, each dog was left for 30 min in the investigation room to accommodate to the environment and operators. To avoid the dog becoming distracted during EEG acquisition, the experiment was performed in a quiet room and no other individuals other than designated operators were allowed inside.

For each patient, a ten minute EEG with an 8-channel examination was recorded for each type of electrode. The electrodes were placed according to previously described protocols [8,25] as follows: FP1, FP2, C3, C4, T3, T4, O1 and O2 in monopolar montage with reference electrodes (A1, A2) placed on the corresponding ear. The needle electrodes were positioned subcutaneously. The disk electrodes were placed in the same position and fixed with a conductive paste (Elefix paste for EEG). All EEG examinations were performed in vigil status (no chemical constraints were used).

To exclude the influence over EEG traces of the same sequence of electrode placements, the order of examination (first needle electrode then disk/first disk then needle) was randomized. The positioning of the electrodes was made each time by the same operator and the correctness was verified by a second operator.

### 2.4. EEG Processing

After a visual assessment of the background, 3 artifact-free epochs of 20 s were selected for each dog and each type of electrode for further processing. In total, 60 datasets (30 for needle electrodes and 30 for disk electrodes) were analyzed. Interhemispheric coherence and correlations were assessed by applying cross scheme 8 (Fp1–Fp2, C3–C4, T3–T4, O1–O2) for each epoch. The dominant frequency (DF) and mean of coherence (MIC) were automatically generated by the software for each frequency band (delta: 0–4 Hz, theta: 4–7 Hz, alpha: 7–13 Hz, low frequency beta: 13–20 Hz and high frequency beta: >20 Hz).

A 0.4 value of coherence (which range between 0—no coherence up to 1—maximal coherence) was established as a threshold.

### 2.5. Statistical Analysis

Data are expressed as mean and standard deviations with maximum and minimum values. After Kurtosis and Skewness test of normality, Mann–Whitney U test and Kruskal Wallis analysis were chosen to compare variables inside and between groups. Values of *p* lower than 0.05 were considered statistically significant. All statistics were performed with the SPSS IBM vs. 17 package program.

## 3. Results

Twenty EEG traces (ten with needle electrodes and ten with disk electrodes for each dog) were recorded and analyzed.

**Figure 1:**
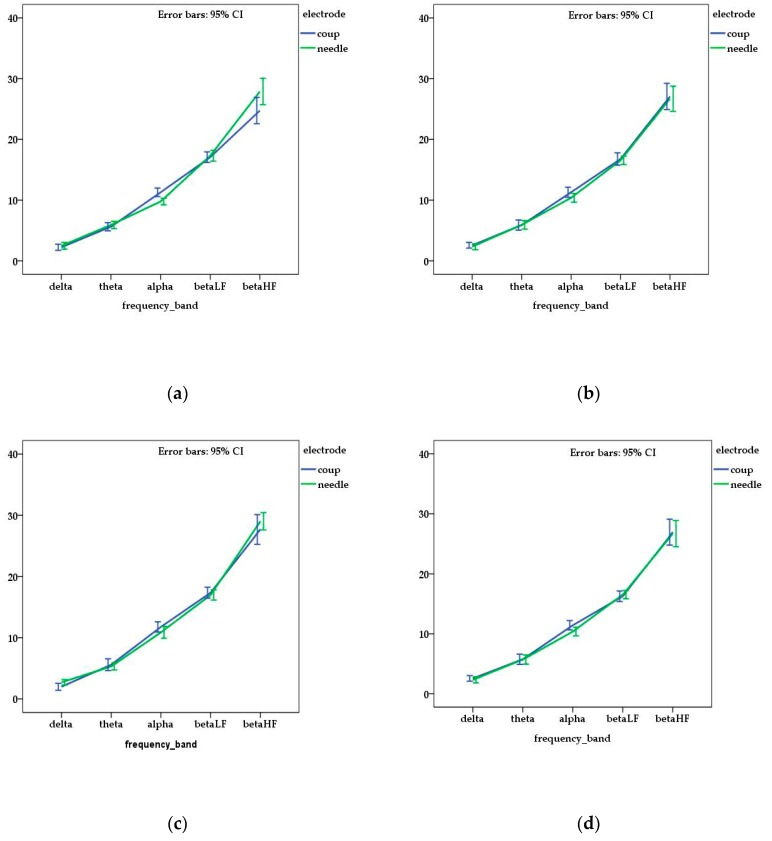
Mean and standard error of dominant frequency for each channel ((**a**) frontal—FP; (**b**) central—C, (**c**) temporal—T; (**d**) occipital—O) and frequency bands related with the type of electrode used in the electroencephalography (EEG) acquisition. No statistical differences (*p* > 0.05) were observed between the obtained values of DF (dominant frequency).

When DF was analyzed in each frequency band, no differences were noted between individuals (*p* > 0.05). In addition, when the DF of each frequency band was compared in relation to the acquisition protocol for each dog, no significant differences were noticed (*p* > 0.05) (Figure 1).

However, when the means of DF of each frequency band were compared between the type of electrodes, statistical differences were observed for FP channel for alpha (*p* = 0.001) and beta HF (*p* = 0.011) waves and for T channel for delta waves (*p* = 0.046). The mean values of DF are presented in Table 1.

Regarding the mean interhemispheric coherence (MIC), the highest value observed was 0.78, with only a few others that ranged between 0.4 and 0.6. The mean and standard deviation of MIC values obtained for all individuals are presented in Table 2.

Statistical differences (*p* < 0.05) were noticed when MIC values were compared for each dog in relation to the type of electrode used. Furthermore, when MIC values were compared for each type of electrode for the entire dog group, significant differences were observed (*p* < 0.05).

**Figure 2:**
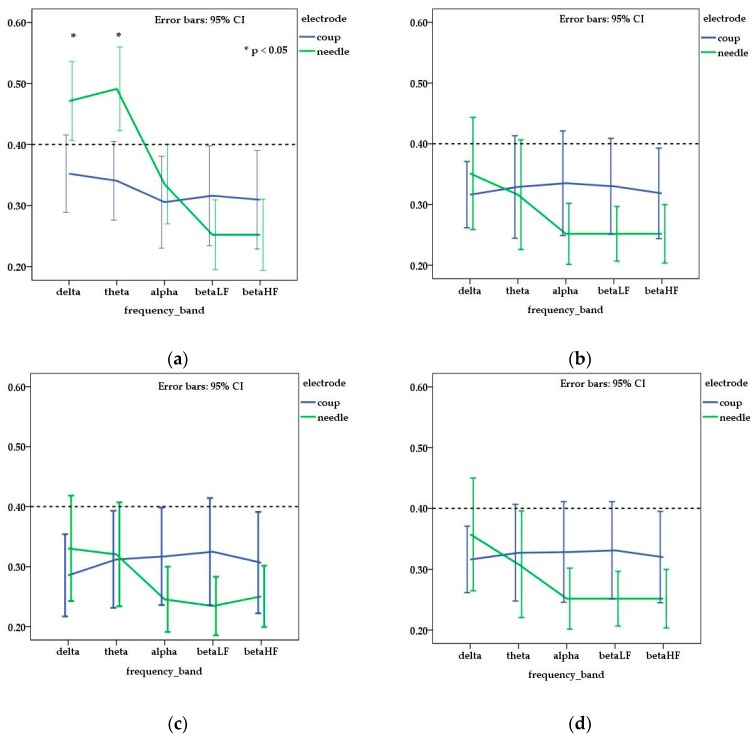
Mean and standard error of interhemispheric coherence observed for needle and disk electrodes in each channel ((**a**) frontal—FP; (**b**) central—C, (**c**) temporal—T; (**d**) occipital—O) and frequency bands. Dotted line (threshold for medium coherence) is fixed at 0.4 corresponding to a medium coherence. The value of obtained coherence was higher than 0.4 in frontal channel for delta and theta frequency bands only on EEG traces acquired with needle electrodes.

When mean values of MIC were compared in relation to the electrode type, a higher coherence was observed for needle electrode traces in FP channel for delta (*p* = 0.006) and theta (*p* = 0.001) frequency bands. In the alpha and beta frequency bands, the coherence values were higher in traces acquired with disk electrodes and, when compared with needle electrodes (Figure 2), significant differences were obtained for C channel in the alpha and beta LF frequency bands (*p* = 0.039 and 0.020 respectively).

## 4. Discussion

The goal of our study was to characterize interhemispheric coherence in vigil dogs when needle and disk electrodes are used for recording EEG traces. To our knowledge, there are no studies in which cerebral coherence is assessed in vigil dogs relative to the type of electrode used during EEG acquisition. Our study shows that the type of electrode influences the registered values of interhemispheric coherence.

Previous research has described data obtained for EEGs in vigil dogs [26] and sedated individuals [8,25]. Brain frequency and coherence were studied in anesthetized dogs [15], and in both healthy and neurological patients [27]. In the present study, we did not find any differences in the background activity and DF between the EEG traces acquired either with needle or disk electrodes. Our results are similar to those previously described by James (2011).

Regarding cerebral coherence in chemically restrained dogs, when needle electrodes were used, a higher interhemispheric coherence (>0.6) was observed in the dorsal channel (FP, P, O) for theta and alpha frequency bands [15]. In our study, the mean of coherence was lower than 0.6 in all the analyzed channels. These results might be a consequence of the fact that we recorded EEG traces only on vigil patients. In the vigil status, coherence is not necessarily predominant in one single frequency band, but different grades of coherence in multiple frequency bands may coexist [14]. In dogs, a coherence value superior to 0.4 was observed only in the FP channel, mainly in the delta and theta frequency bands, when the EEG traces were recorded using needle electrodes. A statistically different value was obtained also for the C channel in the alpha and beta LF frequency bands, but, in those cases, the mean of coherence was inferior to the threshold value (0.4) of the study. This observation is surprising and suggests that, for lower frequency bands, in the FP channel, registered values of the coherence might be dependent on the type of electrode used in the acquisition process.

Our results might have important consequences in the field of canine neurology and applied neuroscience, as frontal channel analysis is preferred in aging and behavior studies. In humans, it is considered that delta oscillations are involved in many cognitive processes [28] and theta waves in the frontal lobes are an indicator of an attentive status. In consequence, cognitive dysfunctions will change the cerebral coherence when assessed using the EEG technique [29]. In dogs, a decrease in total brain volume occurs only in geriatric individuals (12 years and older), but frontal lobe atrophy appears two to four years earlier (8–11 years old). In consequence, the functions related of this region are compromised early in aging [30]. Canine cognitive dysfunction syndrome is usually diagnosed based on imaging appearance, clinical signs, and detection of some metabolites in the cerebrospinal fluid. The clinical signs displayed by affected dogs include disorientation, a decrease in social interactions, changes in sleep–wake cycles, a loss of prior housetraining, increased anxiety, and changes in level of activity [31]. Similar to humans, qEEG may be used as a noninvasive way to determine in a more objective manner, in vigil dogs, the onset and progression of this condition through the assessment of cerebral coherence (especially in the frontal lobes). Therefore, in the light of this study, stainless needle electrodes may be more suitable than the silver disk electrodes in the evaluation of frontal lobe interhemispheric coherence.

Although in our study we observed a net segregation of the obtained coherence values between the frequency bands in the FP channel in relation to the electrode types, we acknowledge some limitations. The study was performed on a limited number of subjects. It is possible that by increasing the number of animals, a higher level of coherence might appear in some frequency bands, different to those presented in our study. There is also a lack of reference values for coherence mapping in vigil dogs, which means that intra- or interbreed brain activity coherence variations must be considered. Finally, our EEG study was performed only on mesocephalic dogs. Our results are not necessarily applicable for dogs with different skull shapes (brahicephalic or dolicocephalic ones).

## 5. Conclusions

In this paper we compared the registered values of interhemispheric cerebral coherence when needle and disk electrodes were used in mesocephalic vigil dogs. Sixty datasets (30 for needle electrodes and 30 for disk ones) were analyzed for dominant frequency and interhemispheric coherence in cross scheme 8 at a 0.4 threshold, established as medium coherence. First, the type of electrode used during EEG acquisition had no influence over the DF values of each frequency band in each individual (*p* > 0.05). Second, the registered values of coherence might be dependent on the type of electrode used in the acquisition process. Thus, the MIC was significantly higher (*p* > 0.05) in the FP channel for the delta and theta frequency bands when stainless needle electrodes were used. Therefore, our results may influence the field of applied neuroscience and canine neurology as the frontal channel analysis is preferred in aging and behavior studies.

Although the results show that the type of electrode influences the registered value of interhemispheric coherence, this paper focuses only on mesocephalic dogs. Thus, the impact of the electrode type on interhemispheric coherence in dogs with different skull shapes (brachicephalic or dolicocephalic ones) must be considered.

## Figures and Tables

**Table 1:** Mean of dominant frequency in all frequency bands relative to the electrode.

Frequency Band	Channel	Disk (mean ± SDev)	Needle (mean ± SDev)	*p* Value
Delta	FP1FP2	2.207 ± 0.997	2.185 ± 1.250	0.941
	C3C4	2.423 ± 0.940	2.493 ± 1.188	0.569
	O1O2	2.331 ± 1.170	1.973 ± 1.149	0.222
	T3T4	2.940 ± 1.061	2.724 ± 1.157	**0.046**
Theta	FP1FP2	5.384 ± 1.192	5.895 ± 1.268	0.113
	C3C4	5.727 ± 1.512	5.824 ± 1.409	0.695
	O1O2	5.598 ± 1.532	5.488 ± 1.317	0.491
	T3T4	5.813 ± 1.689	5.450 ± 1.220	0.214
Alpha	FP1FP2	10.992 ± 1.458	9.672 ± 1.444	**0.001**
	C3C4	11.111 ± 1.945	10.507 ± 1.834	0.234
	O1O2	11.201 ± 1.764	11.232 ± 1.671	0.801
	T3T4	11.489 ± 1.716	10.781 ± 2.080	0.158
Beta LF	FP1FP2	17.332 ± 1.798	16.918 ± 1.771	0.280
	C3C4	16.436 ± 1.913	16.636 ± 1.683	0.604
	O1O2	16.808 ± 1.700	17.037 ± 1.471	0.717
	T3T4	17.211 ± 1.617	16.273 ± 2.265	0.099
Beta HF	FP1FP2	24.360 ± 3.649	27.305 ± 4.674	**0.011**
	C3C4	26.701 ± 3.842	26.818 ± 3.880	0.836
	O1O2	27.432 ± 4.495	26.515 ± 3.874	0.548
	T3T4	27.854 ± 4.355	28.962 ± 3.400	0.428

**Table 2:** Mean interhemispheric coherence in all frequency bands relative to the electrode.

Frequency Band	Chanel	Disk Coherence (mean ± SDev)	Needle Coherence (mean ± SDev)	*p* Value
Delta	FP1FP2	0.372 ± 0.134	0.504 ± 0.197	0.006 *
	C3C4	0.332 ± 0.136	0.349 ± 0.199	0.631
	O1O2	0.309 ± 0.119	0.336 ± 0.179	0.700
	T3T4	0.297 ± 0.150	0.323 ± 0.172	0.620
Theta	FP1FP2	0.371 ± 0.128	0.527 ± 0.169	0.001 *
	C3C4	0.361 ± 0.169	0.335 ± 0.213	0.610
	O1O2	0.321 ± 0.144	0.302 ± 0.167	0.530
	T3T4	0.307 ± 0.140	0.354 ± 0.180	0.183
Alpha	FP1FP2	0.311 ± 0.133	0.347 ± 0.133	0.249
	C3C4	0.331 ± 0.148	0.253 ± 0.118	**0.039**
	O1O2	0.326 ± 0.149	0.263 ± 0.110	0.113
	T3T4	0.309 ± 0.141	0.278 ± 0.155	0.307
Beta LF	FP1FP2	0.299 ± 0.144	0.254 ± 0.104	0.264
	C3C4	0.320 ± 0.143	0.237 ± 0.096	**0.020**
	O1O2	0.317 ± 0.141	0.248 ± 0.095	0.082
	T3T4	0.301 ± 0.150	0.253 ± 0.111	0.329
Beta HF	FP1FP2	0.277 ± 0.139	0.256 ± 0.100	0.716
	C3C4	0.299 ± 0.134	0.240 ± 0.091	0.058
	O1O2	0.292 ± 0.134	0.256 ± 0.095	0.299
	T3T4	0.279 ± 0.145	0.257 ± 0.097	0.733

*—channels in which the mean of coherence was superior to the threshold of 0.4.
